# An Essay on Hernia

**Published:** 1860-03

**Authors:** 


Art. V.—*An Essay on Hernia.* By James Bryan, M.D., Professor of Anatomy in the New York Medical College, and formerly Professor of Surgery in the Medical Colleges of Castleton, Geneva, and Philadelphia. 4to., pp. 14. Philadelphia: F. I. Pilliner, 1860.

We have not had time to examine this work sufficiently to justify us in offering a critical opinion respecting its merits. That the author is well qualified for his task is evinced by the fact that he has been in the profession, in active practice, for upwards of a quarter of a century, and that he has for many years been a teacher of anatomy and surgery. The present number comprises fourteen pages of letter-press, accompanied by a magnificent plate illustrative of the anatomy of inguinal hernia. Four more fasciculi are to complete the work, one being promised every month, at the low price of fifty cents.

